# Introductory Addresses

**Published:** 1889-10-12

**Authors:** 


### THE WINTER SESSION, 1889-90.

### INTRODUCTORY ADDRESSES.

We are not particularly enamoured with the system of inaugural addresses, which obtains at most of the medical schools, although, indeed, several have omitted this part of the initial programme of the winter session, substituting an "old students' dinner," or even allowing the cadets to assemble without any initiatory ceremony. The introductory address, however, allows an opportunity for the chosen speaker to air his pet subjects and ideas, and so, notwithstanding the minimum of utility, the system will die hard, even if it ever altogether succumbs. And as there is at least no harm in this part of the programme, there is as little to be said against it as in favour of it. Last week, following the usual custom, most of the medical schools, both in the metropolis and in the provinces, commenced the winter session of 1889 by the time-honoured introductory address.

